# Contribution of monocyte subpopulations to the pathogenesis and therapy outcome of type 1 diabetes management in pediatric patients

**DOI:** 10.3389/fimmu.2026.1752682

**Published:** 2026-08-05

**Authors:** Aleksandra Starosz, Milena Jamiołkowska-Sztabkowska, Sandra Murawska, Barbara Głowińska-Olszewska, Artur Bossowski, Kamil Grubczak

**Affiliations:** 1 Department of Regenerative Medicine and Immune Regulation, Medical University of Bialystok, Bialystok, Poland; 2 Department of Pediatrics, Endocrinology, Diabetology with Cardiology Divisions, Medical University of Bialytsok, Bialystok, Poland

**Keywords:** immune modulation, monocyte subsets, pediatric diabetes, therapy outcome, type 1 diabetes

## Abstract

Type 1 diabetes mellitus (T1D) is an autoimmune disease characterized by the destruction of pancreas β cells causing impairment in insulin secretion. Despite various factors contributing to T1D, i.e., immunological, genetic, and environmental factors, the exact pathomechanism of T1D remains still not fully understood. Monocytes, the heterogeneous population of immune cells, are the main stones in the innate immune mechanisms. Recently, monocytes have also been considered to be involved in promoting autoimmune disease. Here, our study aimed to evaluate the frequencies of monocyte subsets derived from peripheral blood mononuclear cells (PBMCs) in newly diagnosed pediatric T1D patients at the time of admission. Moreover, our study was followed by a simultaneous assessment of the association of immune parameters with clinically relevant data and disease outcomes. Firstly, we indicated the increased frequencies of intermediate monocytes in T1D patients compared with HC. Those monocytes also presented the increased expression of CD163 activation molecules. Furthermore, the frequency of intermediate monocytes was associated with the GADA autoantibody and the c-peptide levels. Initially increased percentages of intermediate monocytes were followed with higher changes for achieving the clinically relevant c-peptide after 2-year insulin treatment. Interestingly, initial levels of monocyte subsets seem to have no impact on the normalization of HbA1c during treatment; nevertheless, initiative higher frequencies of classical monocytes and lower frequencies of non-classical monocytes were accompanied by higher levels of HbA1c. In conclusion, our findings suggest that monocyte subsets are closely associated with the pathogenesis of T1D and that distinct subsets may have potential value in predicting therapy outcomes.

## Graphical Abstract

**Figure:**
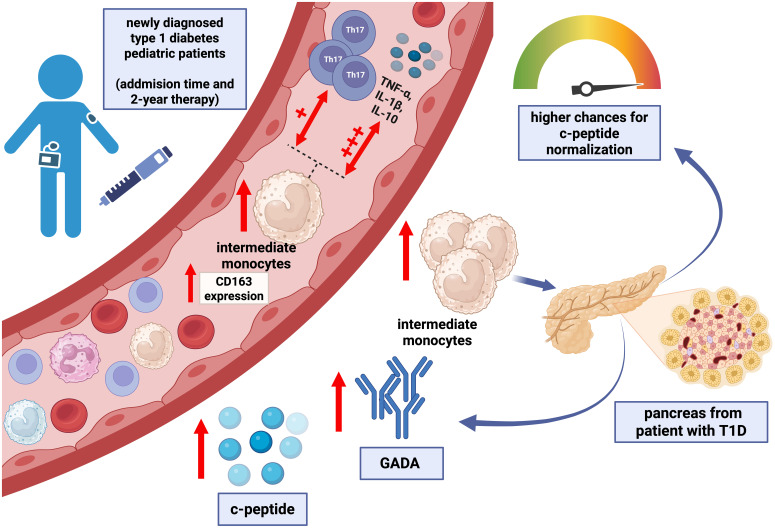

## Introduction

Type 1 diabetes mellitus (T1D) is an organ-specific autoimmune disease characterized by the progressive destruction of insulin-producing βcells in the pancreas, leading to reduced insulin secretion. Children and adolescents are two groups with the highest prevalence of T1D (1). Although various genetic and environmental factors contribute to the condition, the pathomechanism of T1D is not fully understood. The classical paradigm assumes that autoreactive T cells damage β cells in the islets of Langerhans. Consequently, the number of pancreatic cells and their secretory ability decrease, causing an increased requirement for insulin supplementation (2). Chronic hyperglycemia in poorly controlled T1D may lead to severe vascular complications. Interestingly, recent research has indicated an association between elevated levels of CD16+ monocytes and an increased risk of retinopathy development (3). Clinical practice lacks predictors that can be relied on disease monitoring. Despite various factors discovered as crucial in the pathogenesis of T1D, the role of immunological pathways contributing to diabetes shows highest interest, including monocytes (4).

Monocytes are a heterogeneous population of innate immunity cells deriving from the bone marrow. They play a significant role in immune responses to pathogens and prevent excessive tissue damage via phagocytosis, cytokine production, and antigen presentation (5). Interestingly, Martin et al. demonstrated that monocyte infiltration into β cells is sufficient to cause T1D. Increased expression of the chemokine CCL2 in the pancreatic β cells led to the recruitment and accumulation of monocytes in mice models (6). Additionally, abnormal NF-κB signaling reported in monocytes and dendritic cells is considered a relevant factor in T1D pathogenesis. Impaired dendritic cells (monocyte-derived) may inappropriately activate T cells and as well as contribute to autoaggressive processes (7). Furthermore, previous research demonstrated that monocytes from T1D patients can induce Th17 cells, which are crucial in promoting autoimmunity (8).

Monocytes consist of three distinctive subsets: classical (CD14++CD16−), intermediate (CD14++CD16+), and non-classical (CD14+CD16++) monocytes (9). In healthy individuals, peripheral monocytes consist of approximately 80%–90% classical, 5%–10% intermediate, and 2%–10% non-classical subsets (10). Classical monocytes are characterized by strong phagocytic activity, high expression of CD14 (a pathogen recognition receptor), and secretion of proinflammatory cytokines such as IL-6 and IL-8 (11). Moreover, some articles linked increased levels of IL-6, IL-1β, and TNF-α to an increased monocyte frequency (4, 11). Conversely, non-classical monocytes are involved in the vascular steady state of inflammation and promote neurovascular repair (12). Elevated levels of these monocytes were observed in other autoimmune diseases, including systemic lupus erythematosus and Hashimoto’s thyroiditis (13). Intermediate monocytes participate in antigen presentation to T cells (through increased MHC class II molecule expression), and preserve proinflammatory abilities via TLR2 and TLR4 (14). Recent investigations reported a significant role of these monocytes in promoting inflammatory responses in T1D (14, 15). Increasing evidence indicates that shifts in the distribution and functional state of monocyte subsets accompany chronic inflammatory diseases and reflect ongoing immune activation. In T1D, recent studies have demonstrated an expansion of inflammatory intermediate monocytes and increased pro-inflammatory activity, both of which correlate with residual β-cell function and clinical disease progression. These observations support the evaluation of circulating monocyte subsets as potential biomarkers of immune dysregulation in the early stages of T1D (16, 17).

CD163 is a membrane-bound scavenger receptor of the SRCR family, mainly expressed on monocytes and macrophages. In healthy individuals, nearly all circulating monocytes express CD163, which acts as the primary receptor for the hemoglobin–haptoglobin complex (Hb-Hp), mediating its clearance and protecting against oxidative damage (18). Furthermore, CD163 exerts a well-recognized anti-inflammatory and immunoregulatory role, with its expression reflecting the activation state and polarization of monocytes/macrophages toward an M2-like (anti-inflammatory) phenotype. The soluble form, sCD163, is released during activation of monocytes/macrophages and is widely regarded as a circulating biomarker of immune activation and metabolic stress (19, 20). Therefore, the evaluation of CD163 expression on monocytes should be essential for determining early alterations in their anti-inflammatory and immunoregulatory profiles in newly diagnosed T1D patients.

To date, there are still gaps in our knowledge regarding the role of monocytes in the onset and progression of type 1 diabetes. Our study aimed to evaluate the frequency of monocyte subsets in newly diagnosed pediatric T1D patients at admission time. Subsequent assessment will be followed by an assessment of the association between immune parameters and clinical manifestations and possible ongoing disease outcomes.

## Materials and methods

### Patients’ characteristics

The study design comprised 59 pediatric patients with newly diagnosed T1D pediatric and 24healthy controls (HC). All patients were enrolled to the study at the Department of Pediatrics, Endocrinology, Diabetology with Cardiology Division, University Children’s Teaching Hospital Bialystok. The control group consisted of children referred for cardiological evaluation because of heart murmurs, incidental syncope, or other cardiovascular symptoms requiring further diagnostic work-up. Following clinical assessment, no significant cardiovascular disease was diagnosed, and diabetes, inflammatory disorders, autoimmune diseases, and other metabolic conditions were excluded prior to study enrollment. Patients were diagnosed in accordance with the ISPAD guidelines and initially received intravenous insulin therapy. Once their condition stabilized, the treatment was changed to subcutaneous functional intensive insulin therapy, administered either as multiple daily injections or via continuous subcutaneous insulin infusion. Blood was collected from metabolically stable patients, not in acute acidosis, before initiating the therapeutic regimen. The patients’ characteristics have been attached (**Supplementary Table 1A**). Informed consent was obtained from individual subjects (or their parent or legal guardian for underage subjects). The Ethical Committee at the Medical University of Bialystok approved the study protocol: APK.002.22.2021.

Biochemical and serological parameters measured during routine diagnostics and used for T1D patients’ selection were as follows: venous glycemia >200 mg/dL, clinical manifestation, presence of autoantibodies—GADA, IA2A, ICA. Additional assessments included body mass index (BMI), blood glucose levels (glycemia), glycated hemoglobin (HbA1c), fasting c-peptide, and daily insulin requirement (DIR; insulin/kg/day). Therapy was monitored using HbA1c, DIR, and c-peptide at selected time points: 3 months, 6 months, 1 year, 2 years. Remission was defined as DIR (<0.5 units/kg/day) and HbA1c (<7%). Biochemical and serological data from the T1D group were included (**Supplementary Table 1B**). Follow-up data at the final study visit were available for 53 participants; loss to follow-up was primarily due to relocation or other logistical reasons unrelated to disease characteristics or study procedures.

### Research material

4.9 ml of EDTA-anticoagulated peripheral blood was collected by venipuncture at the time of admission. First, plasma was obtained by centrifugation of the full blood at 400 g, 10 min, with second centrifugation at 1,810g, to exclude morphotic elements. Peripheral blood mononuclear cells (PBMCs) were isolated using gradient density centrifugation on Pancoll 1.077 g/L gradient (PAN Biotech) and centrifugation at 1,250g, 25 min (slow acceleration/deceleration). PBMCs were washed in phosphate-buffered saline (PBS; Corning) and preserved viable in liquid nitrogen using cryoprotectant fetal bovine serum (FBS; PAN Biotech) with 10% DMSO (Sigma-Aldrich). PBMCs were gradually cooled (1 °C per minute) using a controlled-rate freezing container placed in a −80 °C freezer. After 2 weeks of storage at −80 °C, the samples were transferred to a liquid nitrogen container for long-term preservation until analysis.

### Flow cytometric evaluation of monocytes

Preserved PBMCs were first rapidly thawed and resuspended in the culture medium (RPMI 1640 + 10% FBS). The obtained cells’ number was calculated using the hemacytometer of the Bürker chamber and stained with the 0.4% trypan blue solution (Sigma-Aldrich) to establish the cells’ viability. PBMC viability was assessed before flow cytometric analysis as a quality-control measure. Only samples with viability between 96% and 100% were analyzed. All samples in the study met this criterion, and no participants were excluded based on PBMC viability. The flow cytometric assessment was performed using 250,000-500,000 cells, with acquired data normalized to include eventual differences in the initial cell numbers. PBMCs were stained with the monoclonal antibodies conjugated to fluorochromes: anti-CD14 PerCP (clone MφP9), anti-CD16 FITC (clone 3G8), anti-CD163 PE (clone GHI/61) (BD Biosciences). Following incubation, unbound antibodies were washed out with PBS, and the remaining cells were suspended in fixation buffer (BD Biosciences). Data were acquired using the FACSCalibur flow cytometer (BD Biosciences, San Jose, CA, USA) and processed with FlowJo software (Tree Star Inc., Ashland, OR, USA). First, the morphology of monocytes was determined using relative size (forward scatter, FSC) and granularity/complexity (side scatter, SSC). The CD14 surface marker was applied to confirm monocyte phenotype. Three monocyte subpopulations were distinguished based on differential expressions of CD14 and CD16: classical (CD14++CD16−), intermediate (CD14++CD16+), and non-classical (CD14+CD16++). Within each subset, the expression of the CD163 activation marker was analyzed. The gating strategy, together with the proper controls applied, is attached (**Supplementary Figure 1**).

### Immunoenzymatic cytokines analysis

Plasma samples were used to detect selected cytokines. Immunoenzymatic DuoSet ELISA Kits (R&D Systems) were used in accordance with the manufacturer’s protocols. The following cytokines were analyzed: sCD163 (LOD = 156–10,000 pg/ml), TNF-alpha (LOD = 15.6–1,000 pg/ml), IL-1beta (LOD = 3.91–250 pg/ml), and IL-10 (LOD = 31.3–2000 pg/ml). The absorbance level was measured at a 450-nm wavelength using a Ledetect96 microplate reader (Labexim Products, Lengau, Austria). The standard curve (four-parameter logistic) allowed for calculation of the tested cytokine concentrations.

### Statistical analysis

Statistical processing was performed using GraphPad Prism 9.0 software (GraphPad Prism Inc., San Diego, CA, USA). Based on the data’s non-Gaussian distribution, the non-parametric Mann–Whitney U test was used to assess differences between groups. The Wilcoxon test allowed determination of statistically significant changes over the course of T1D patients’ therapy. Median values with interquartile range were presented on the graphs. The significance level was set at a p-value of 0.05. Correlations were determined using a nonparametric Spearman test, presented as heat maps.

## Results

### Monocyte subsets frequency in diabetic patients and correlation with clinical parameters

**Figure 1:**
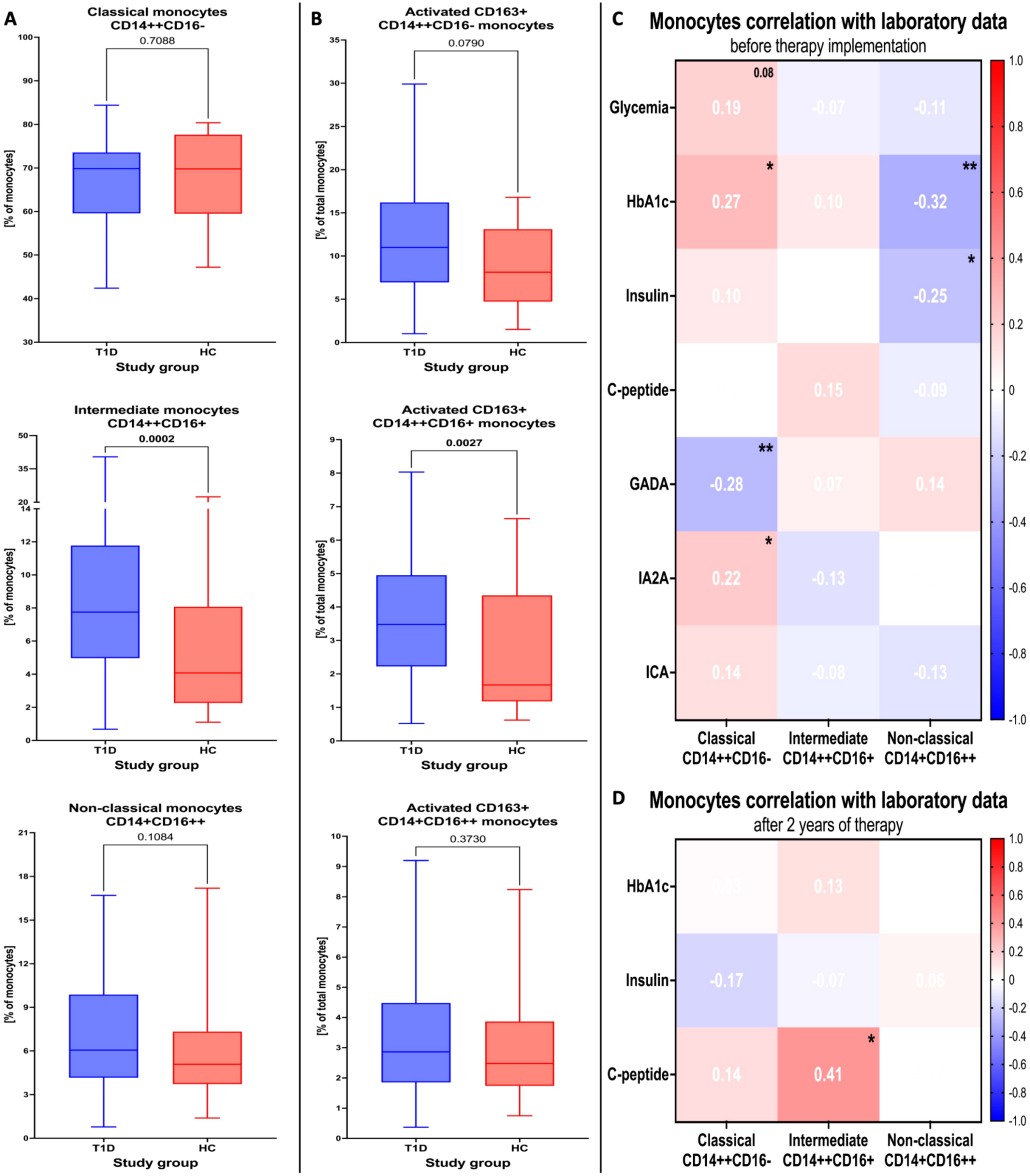
Variations in monocyte subsets’ frequency **(A)**, including CD163+ cells **(B)**, within type 1 diabetes (T1D) and healthy control group (HC). Correlations of monocyte subsets with clinical laboratory parameters in type 1 diabetic patients, at admission **(C)** and after 2 years of the therapy **(D)**. Presentation of exact r values on heat-maps. The significant values are demonstrated with asterisks or p values: * p<0.05; ** p<0.01. Correlation strength was grouped on the basis of the coefficient values: weak (r = 0.20-0.39), moderate (0.40-0.59) and strong (0.60-0.80).

First, we determined that the initial frequencies of intermediate monocytes were significantly higher in T1D patients than in the HC group. Classical and non-classical monocytes showed no differences between analyzed groups. (**Figure 1**) Next, we noticed that the level of activated CD163+ intermediate monocytes was also significantly elevated in pediatric T1D patients. Additionally, a slight tendency for higher levels of CD163+ monocytes was also observed in a subset of classical monocytes. (**Figure 1**) We noticed that HbA1c, together with IA2A autoantibodies are positively correlated with classical monocytes at the recognition stage. In contrast, GADA autoantibodies showed negative association with that subset. Non-classical monocytes were negatively correlated with HbA1c levels and daily insulin requirement (DIR) at admission. (**Figure 1**) Subsequent monitoring revealed a positive correlation between intermediate monocytes and c-peptide after 2 years. (**Figure 1**) Considering activation status of monocyte subsets (expressing CD163), no statistically significant associations with diabetes-related parameters were found, neither at admission nor following treatment. (**Supplementary Figure 2**).

### Diabetic related parameters may influence on the monocyte subsets frequencies in T1D patients

**Figure 2:**
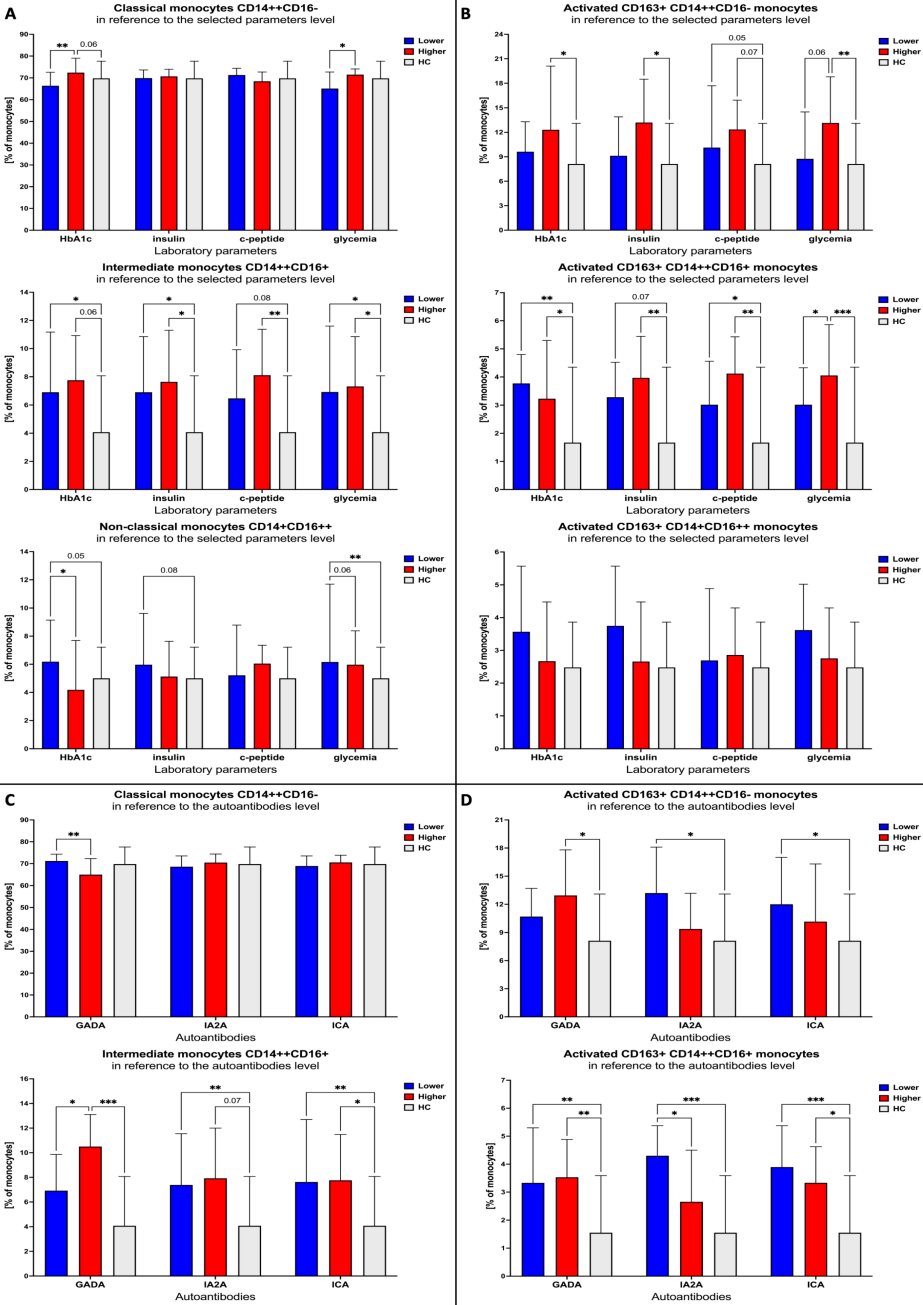
Variations in monocyte subsets frequency in T1D patients, including diabetic-related biochemical parameters stratification into lower/higher levels **(A)**, including activated CD163+ monocytes analysis **(B)**. Separate assessment based on serological data (GADA, IA2A, ICA) was performed in monocyte subsets **(C)** and those CD163+ cells **(D)**. The significance levels indicated with asterisks or exact p values: * p<0.05; ** p<0.01; *** p<0.001.

We further stratified diabetic patients on the basis of the median values (lower/higher) of clinical parameters (HbA1c, DIR, c-peptide, or glycemia). T1D patients with higher initial values of HbA1c and glycemia showed elevated frequencies of classical monocytes at the admission. In contrary, diabetic subjects with lower HbA1c or glycemia had increased levels of non-classical monocytes. Low/high levels of selected laboratory parameters had no substantial association on equally high intermediate monocytes in T1D patients compared with the control group. (**Figure 2**) Elevated levels of activated CD163+ classical monocytes, compared with the control group, seemed to be exclusively correlated with higher HbA1c, DIR, and glycemia. Higher median glycemia values were also associated with increased numbers of intermediate monocytes expressing CD163, even among T1D patients. No significant correlations were observed between diabetic laboratory parameters and CD163+ non-classical monocytes (**Figure 2**). In the context of T1D-specific autoantibodies (GADA, IA2A, and ICA), patients with higher initial levels of GADA showed a lower frequency of classical monocytes. On the other hand, elevated percentages of the intermediate subset were reported in diabetic subjects with higher GADA, with no effect of IA2A or ICA on those cells. A similar tendency was observed in context of non-classical monocytes. Classical monocytes were found to be increased in T1D patients with initially lower GADA levels. (**Figure 2**) Intermediate monocytes with CD163 in T1D had equally increased frequencies compared with the control group, irrespective of lower/higher values of tested autoantibodies. Interestingly, diabetic subjects with lower IA2A or ICA had significantly higher classical monocytes versus healthy controls. (**Figure 2**) The frequency of CD163+ non-classical monocytes were not significantly correlated with autoantibody status. (**Supplementary Figure 3**).

### Association of monocyte levels on the laboratory parameters in the course of therapy

**Figure 3:**
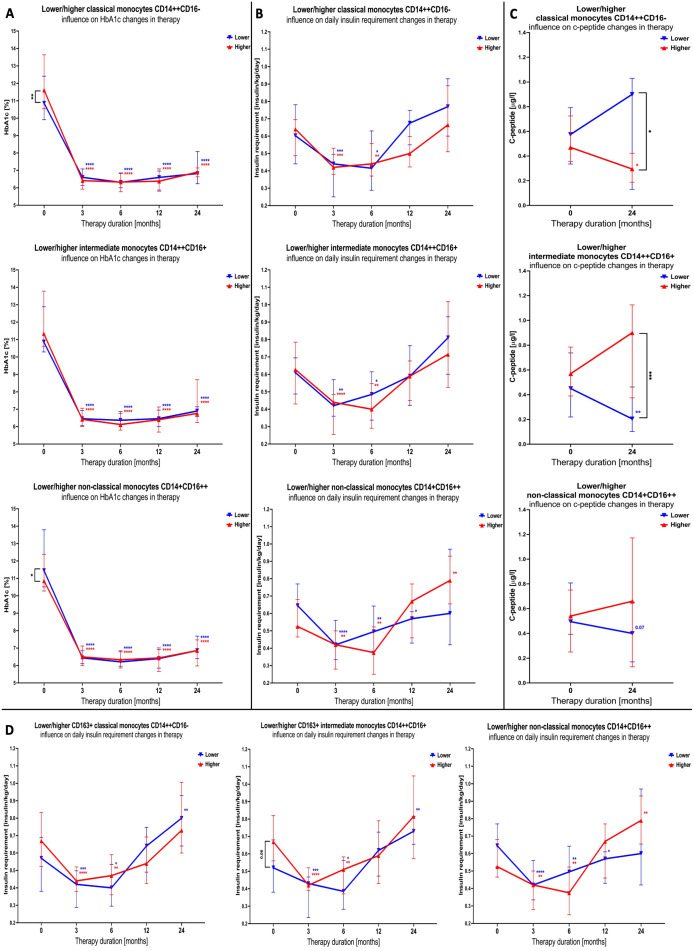
Levels of monocyte subpopulations influence the changes within selected parameters in the course of the T1D therapy. Variations in time of admission were reported in the context of HbA1c **(A)**, insulin requirement **(B)**, and c-peptide **(C)**, within T1D low/high monocyte groups. Effect of CD163+ monocyte subset levels on the changes within insulin requirement in the course of the T1D therapy. Significant differences were distinguished with asterisks or exact p values (color for time-based changes; black brackets for differences between groups): *p<0.05; **p<0.01; ***p<0.001; ****p<0.0001.

Therapy implementation led to a significant decrease in HbA1c, regardless of initial monocyte subset levels. Nevertheless, admission values of HbA1c were significantly higher in T1D patients with higher/lower frequencies respectively of classical and lower non-classical monocytes. (**Figure 3**) Considering daily insulin requirement (DIR), we observed its gradual decrease within 6 months, regardless of initial monocyte subset levels. Subsequent months were followed by an increase of DIR. Noteworthily, at the 2nd year of the management, diabetic patients with baseline elevated non-classical monocytes required significantly higher doses of insulin daily. (**Figure 3**) Regarding c-peptide, pretreatment lower classical monocytes were associated with higher values of that insulin-related parameter in contrast to the opposite group after therapy. Conversely, lower levels of intermediate monocytes were associated with a significant decline in c-peptide concentrations, unlike in subjects with higher levels of these cells. In contrast, CD163+ non-classical monocytes showed no significant correlation with c-peptide levels, although a slight trend toward lower concentrations was observed in the low-monocyte group. (**Figure 3**).

### Influence of activated CD163+ monocyte levels on the laboratory parameters in the therapy

Initial values of HbA1c showed no differences in diabetic subjects considering low/high CD163+ monocyte levels. The therapy-induced decline in HbA1c was not related to monocyte subset activation status. Interestingly, at the 2nd year of T1D management, higher admission levels of intermediate and non-classical monocytes were associated with elevated HbA1c. (**Supplementary Figure 4**) Daily insulin requirement (DIR) seemed to be increased in subjects with higher pretreatment CD163+ intermediate monocytes. DIR gradually decreased in time till the 6th month of therapy, at a comparable rate in all CD163+ monocyte subsets. Further monitoring revealed reversed changes in DIR with its higher values compared with admission in patients with low CD163+ classical and intermediate monocytes. In CD163+ non-classical monocytes, initially higher values of cells were related to higher insulin requirement after the 2-year therapy. (**Figure 3**) The C-peptide did not show significant changes in time, with only a tendency for a decline in high classical and low non-classical CD163+ monocyte patients. (**Supplementary Figure 4**).

### Association of monocyte subpopulation levels at admission with therapeutic outcome in T1D

**Figure 4:**
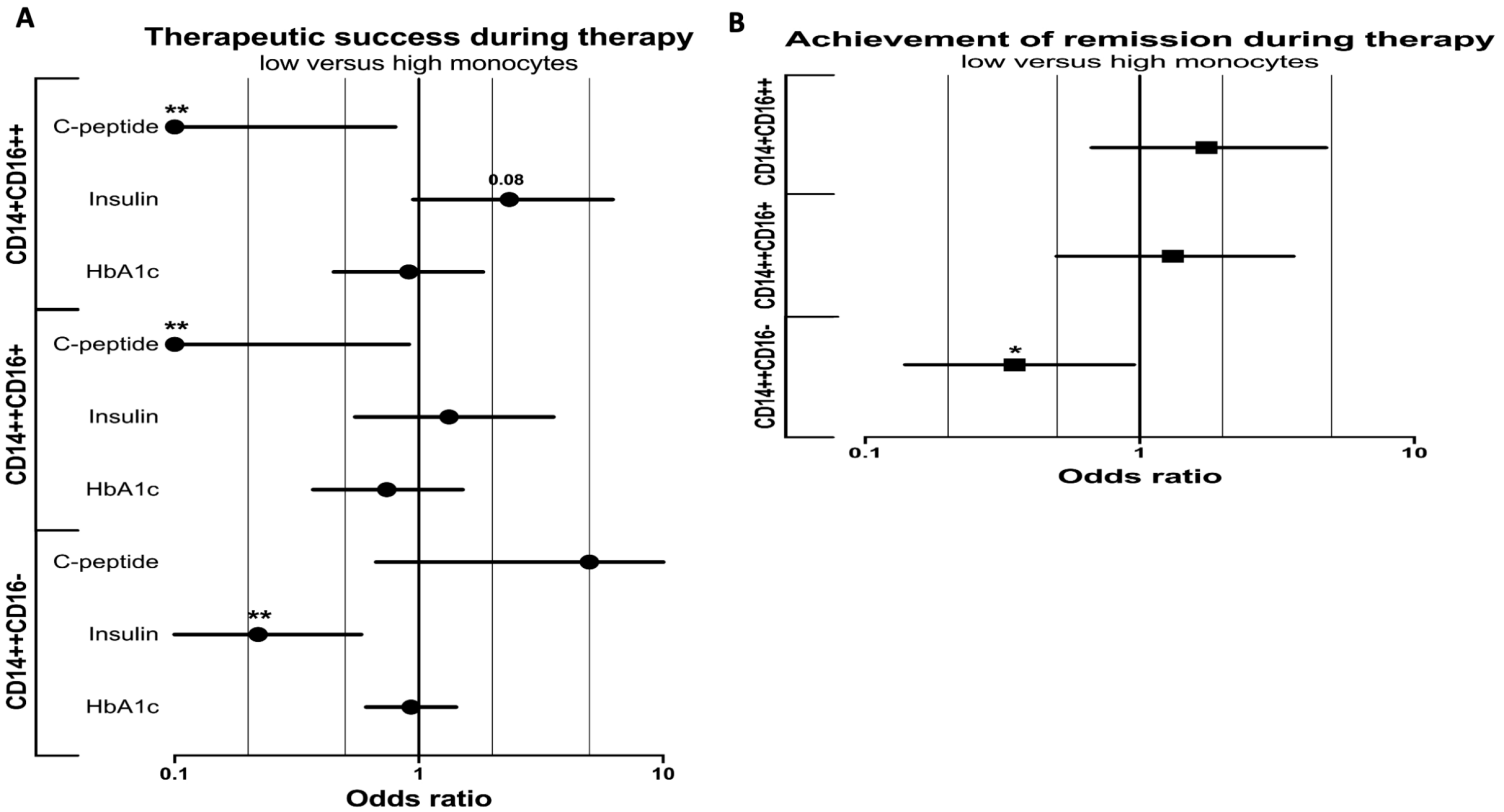
Influence of monocyte subpopulation levels at admission on therapeutic success achievement in T1D after 2-year therapy, based on the normalization of the selected laboratory parameters **(A)** and remission occurrence **(B)**. The significance levels indicated with asterisks or exact p-values: * p<0.05; ** p<0.01.

Odds ratio analysis allowed us to find that lower initial values of non-classical monocytes are associated with low chances of therapeutic success measured as c-peptide preservation after 2 years. The same effect was observed with intermediated monocytes. Contrarily, low baseline non-classical monocytes showed a tendency for increased chances for insulin requirement stabilization after therapy. In the case of classical monocytes, low levels were linked to lower odds for insulin usage normalization. (**Figure 4**) In addition, lower chances for remission were also related to pretreatment low levels of classical monocytes. Baseline frequencies of intermediate and non-classical monocytes showed no statistically significant association with remission occurrence. (**Figure 4**) Evaluation of the odds for parameter normalization or remission achievement demonstrated no significant link with initial levels of monocytes with CD163 expression. (**Supplementary Figures 5A, B**).

### Cytokine profile correlation with the different monocyte subset levels

We showed no significant differences in the sCD163, TNF-α, IL-1β, or IL-10 level between T1D patients and the control. (**Supplementary Figure 5C**) A weak positive correlation of CD163+ intermediate monocytes with TNF-α, IL-1β, and IL-10 was reported. (**Supplementary Figure 5D**) Moreover, based on previously published data on Th17 and Treg lymphocytes in T1D (21), a negative weak correlation of classical monocytes with Th17 cells and a positive weak correlation with intermediate monocytes was found. Non-classical and CD163+ intermediate monocytes were positively associated with regulatory T cells. (**Supplementary Figure 5E**).

## Discussion

Monocytes are one of the most critical elements of the innate immune system. Aside from their immunomodulatory properties and role in tissue regeneration, their activity has been linked to the development of autoimmune diseases (22). Despite the central role of autoreactive B and T cells in diseases like type 1 diabetes (T1D), the function of monocytes might play an essential role in the pathogenesis and, thus, affect clinical outcome (23).

First, we revealed that newly recognized T1D pediatric patients demonstrate a significantly higher frequency of intermediate monocytes, with no differences in classical or non-classical monocytes. That observation was accompanied by higher levels of activated CD163+ intermediate monocytes in diabetic subjects. Similarly, Ren et al. showed increased levels of intermediate monocytes in pediatric diabetes patients. Their observation was followed also by general increasing of the monocytes, not the intermediate subset exclusively (15). Ryba-Stanisławowska et al. presented elevated CD16+ monocytes in T1D, with HbA1c levels were associated with elevated numbers of intermediate and non-classical monocytes (3). Consequently, the chronic inflammatory process might result from the expanded infiltration of CD16+ monocytes, leading to support of an autoreactive response (24).

Through additional stratification of T1D patients, we showed that groups with below-median values of HbA1c and glycemia had reduced frequencies of classical CD14++CD16− monocytes. High levels of HbA1c were positively correlated with the level of classical monocytes. These monocytes are the source of proinflammatory cytokines, including IL-6 and TNF-alpha. Given the positive association between those cytokines and VEGF concentration, high levels of inflammatory monocytes might contribute to an increased risk of microvascular complications (3). Zahran et al. reported an increased frequency of intermediate and non-classical monocytes in pediatric T1D, with a positive association between monocyte levels (classical subsets predominantly) and HbA1c (25). Here, we showed a negative correlation of non-classical monocytes with an HbA1c level, supporting the hypothesis of these cells’ potential role in the early stages of type 1 diabetes in children.

The subset of non-classical monocytes was elevated in patients with lower GADA autoantibodies. On the other hand, intermediate monocytes demonstrated a stronger link to the autoimmune process with elevated frequencies in diabetic patients with higher GADA levels despite no differences in biochemical data. A similar tendency was observed in reference to non-classical monocytes and GADA values; however, there was also a higher frequency of those monocytes in patient group with lower HbA1c values at the same time. To the best of our knowledge, that is the first time the association of the initially higher GADA levels with increased frequencies of intermediate monocytes was reported, thus suggesting the undeniable role of that subset in the autoimmune inflammation accompanying T1D.

Higher median values of all selected biochemical parameters together with elevated classical CD163+ monocytes were revealed in T1D subjects compared with the control group. It was also reported in subjects with elevated GADA autoantibodies. Either classical and intermediate CD163+ monocytes showed elevated frequencies in patients with higher glycemia values. Interestingly, intermediate monocytes expressing CD163 were lower in groups with higher IA2A autoantibodies. The activity of monocytes had no influence on biochemical or serological parameters in reference to CD163+ non-classical monocytes. To date, several studies have indicated the increased islet infiltration by CD163+ M2 macrophages in T2D diabetes. Those are thought to have antidiabetic properties through inhibition of T2D progression, insulin resistance reduction, and beta cell regeneration (26, 27). Notably, the gene expression of CD163 within pancreatic tissue was lower in the pre-diabetic patients compared with the controls (28). Most studies considered monocyte contribution in T1D usually as a total population, including methods of reduction of those cells’ activation and further pro-inflammatory cytokine decline in T cells preventing cytotoxicity on beta cells (29) or recently introduced diagnostic potential, like lymphocyte-to-monocyte ratio (LMR) (30). Here, we demonstrated the significance of monocyte subset determination when investigating their role in diabetes, especially in the context of novel diagnostic or therapeutic methods.

Only minor associations were found between monocytes and diabetes-related parameters. Intermediate monocytes did not show any correlation with laboratory parameters at the admission stage. However, that subset showed a positive moderate association with c-peptide after 2 years of therapy. Before therapy, only weak correlations were demonstrated between classical monocytes and HbA1c, glycemia, and IA2A. A weak negative connection was found between non-classical monocytes and HbA1c or daily insulin requirement (DIR). No significant correlations were reported regarding CD163+ monocytes. Previously, elevated intermediate monocytes were shown in subjects with higher HbA1c. The frequency of intermediate monocytes negatively correlated with insulin concentration or c-peptide, specifically to diabetic patients with remnant pancreas function. In accordance, new light was shed on the potentially destructive effect of intermediate monocytes on residual β cells. Studied intermediate monocytes were also characterized by the increased expression of CD11b and CD62L adhesion molecules, confirming their increased capability to migrate into the inflammation site (15).

T1D therapy was efficient regardless of the low/high admission monocyte subset levels, with expected HbA1c maintained through the study. Notably, higher HbA1c levels were observed before therapy in subjects with higher frequency of classical or lower of non-classical monocytes. Lower/higher frequencies of CD163+ subsets did not affect HbA1c. Some studies indicated decreasing percentages of non-classical monocytes in T2D patients with complications (31). Patients with complicated diabetes demonstrated low CD163+ monocytes, together with high circulating soluble CD163 (sCD163) (32). Lack of glycemic control can accelerate vascular damage and contribute to the cardiovascular complications. Adult patients with T1D are characterized by an increased risk for infections, primarily associated with HbA1c, obesity, and disease duration (33). The glycolytic activity of peripheral monocytes was found to be significantly lower after *in vitro* glucose application, with no differences in baseline metabolism. These results revealed a defective function of monocytes in the presence of high-glucose conditions. Lack of glycemia control, reflected by high HbA1c, was followed by reduced monocyte activity, with decreased TNF-alpha and IL-1beta secretion. Changes in their function were found to have a substantial association with HbA1c but not the disease duration (34).

Macrophages, as tissue-differentiated forms of monocytes, have been shown to affect chronic inflammation and insulin resistance in muscles under high glucose concentrations (35). Here, we extended that area of knowledge with monocyte subset contribution. DIR was reduced within 6 months of treatment, with a further increase later. T1D with elevated initial non-classical monocytes showed even higher DIR at the 2^nd^ year of therapy compared with admission level. Increased CD163+ intermediate monocytes were associated with initially increased requirement for insulin. The obtained data are in consent with a recently published negative association between higher intermediate monocytes and a decline in c-peptide production, leading to elevated insulin requirement (17). In addition, T1D patients with partial remission showed a lower frequency of both CD16+ monocyte subsets (36). The lower pretreatment frequency of CD163-expressing classical and intermediate monocytes was associated with even higher DIR after 2 years. The same pattern was demonstrated for elevated values of non-classical monocytes, leading to increased DIR. Non-classical monocytes seemed to correlate positively with the preserved function beta cells, reflected by c-peptide. Further investigation might be required considering the fact that previous data were obtained on subjects’ samples months after diagnosis (with well-controlled glycemia) and not right at T1D confirmation (17). Our subjects demonstrated the full spectrum of biochemical and clinical features of that diabetes type.

Beyond their role as circulating innate immune cells, monocytes constitute the principal precursors of tissue macrophages, which are critical regulators of immune homeostasis and inflammatory responses. Following recruitment into pancreatic tissue, activated monocytes may differentiate into macrophages that amplify local inflammation through cytokine secretion, antigen presentation, and interactions with autoreactive T lymphocytes, thereby contributing to β-cell destruction. Conversely, macrophages also participate in the resolution of inflammation and tissue repair, highlighting the functional plasticity of the monocyte–macrophage axis. Therefore, alterations in circulating monocyte subsets observed at disease onset may not only reflect systemic immune activation but may also mirror the dynamic processes occurring within the pancreatic microenvironment. These findings support the concept that monocyte subsets represent clinically relevant biomarkers of innate immune activation and warrant further longitudinal investigation to determine whether their temporal changes during insulin therapy could predict disease progression, preservation of residual β-cell function, or the achievement of partial remission (16, 17).

Patients with high intermediate monocytes before therapy showed even slightly elevated c-peptide levels at the 2nd year of monitoring, in contrast to reduced values in the group with low frequency of that subset. It is unknown whether that association results only in preserved insulin production or contributes to some novel functions of c-peptide, including anti-inflammatory and anti-apoptotic influence in T1D (37). Initially elevated classical monocytes were associated with c-peptide reduction during treatment. The question remains: what is the link between tested immune and pancreatic cells? Dekker et al. reported that extracellular vesicles (EVs) released by stress-induced beta cells (EndoC-βH1 cell *in vitro* model) activated the pro-inflammatory phenotype of monocytes and antigen presentation (38). Our results complement those data showing variations in monocyte subset response in T1D patients, suggesting the ambiguous nature of interactions between monocytes and beta islets cells.

We cannot select individual subpopulation of monocytes that has the highest influence on the course of T1D in children, confirming that with analysis of diabetes-related parameter normalization at the end of therapy. Lower initial intermediate and non-classical monocytes were associated with reduced chances for reaching preservation of c-peptide levels. Conversely, decreased classical monocytes could predict a lack of DIR normalization. Importantly, the same monocyte subset was concomitantly linked to reduced chance of remission. CD16-positive monocytes were not significantly related to the remission odds, neither activated CD163+ monocytes were. Villalba et al. demonstrated decreased monocytes at the early stage of T1D in pediatric patients and further decline with the disease progression. This might suggest an active infiltration to tissues and consequently contribution to the long-lasting autoimmune response (39). These results correspond with our finding where a lower initial level of classical monocytes was related to lower chances for disease remission after 2 years.

Recently, selected cytokines showed an association of BMI and SDS-BMI in T1D patients (40). Here, no significance was demonstrated in selected monocyte-related cytokines between T1D and control groups. Nevertheless, CD163+ intermediate monocytes were the only ones showing a positive correlation with TNF-alpha, IL-1beta, and IL-10 cytokines, highlighting the subset’s role as a main source of inflammatory cytokines, presented previously using *in vitro* models (41). Monocytes isolated from T1D adult patients were reported to secrete less proinflammatory cytokines than healthy controls after stimulation (34). Recent studies indicated CD163 as involved in the hemoglobin–haptoglobin complex removal contributing to the anti-inflammatory responses through IL-10 and NO (42). sCD163 can also modulate the immune response via inhibition of T-cell proliferation (43). sCD163 in plasma might be related to the M2 macrophages playing substantial role in tissue repair (44).

Intermediate and non-classical monocyte subsets were found to correlate positively with Th17 and Treg lymphocytes, respectively, whereas classical monocytes showed a negative association with Th17 cells. These relationships provide novel insight into the cooperation between innate and adaptive immune responses during the autoimmune process accompanying T1D (21). The increased frequency of IL-1b+ or IL-6+ monocytes might be associated with greater expansion of Th17 lymphocytes. The previously described mechanism may involve spontaneous secretion of proinflammatory cytokines by classical monocytes, with modulated release of IL-17 from T cells (8). Taken together, our findings indicate that alterations in circulating monocyte subsets are associated with both metabolic and immunological features of newly diagnosed pediatric T1D, supporting their potential utility as accessible biomarkers reflecting innate immune activation at disease onset. Future longitudinal studies are warranted to determine whether dynamic changes in monocyte subsets during insulin therapy may predict preservation of β-cell function, partial remission, and long-term clinical outcome.

Nevertheless, we are aware of several limitations of our study. First, it was conducted on a relatively small cohort from a single center, which may limit the generalizability of the findings and preclude the use of more complex multivariable models to comprehensively adjust for potential confounding factors. Therefore, the reported associations should be considered exploratory and hypothesis-generating. Second, although participants were prospectively followed for 24 months, not all patients completed all follow-up assessments, resulting in missing observations in selected analyses. Missing values were not imputed, and all analyses were performed using available-case data only. Third, analyses were performed on cryopreserved PBMCs rather than freshly isolated blood, preventing the determination of absolute monocyte counts; therefore, results are presented as relative frequencies. Finally, as an observational study, the reported associations between monocyte subsets, CD163 expression, and metabolic parameters cannot be interpreted as causal, and functional studies are required to clarify the biological mechanisms underlying these relationships.

## Conclusion

Despite commonly known causes of hyperglycemia, the exact mechanism of pancreatic β-cell impairment remains unknown. Non-effective treatment with long-lasting hyperglycemia is a substantial risk factor for further cardiovascular complications. Monocytes are considered significant in the promotion of the autoimmune process. We determined that T1D demonstrates elevated intermediate monocyte levels, with activation of these cells associated with initially increased insulin requirement. A higher frequency of classical monocytes and a lower frequency of non-classical monocytes were associated with higher HbA1c. Noteworthily, intermediate and non-classical monocytes were associated with reduced chances of achieving c-peptide normalization. Cumulatively, we showed the crucial contribution of monocyte subsets to T1D, together with predictive value in therapy outcome. Further mechanistic and longitudinal studies are of great importance in validating those monocyte subsets’ causal role in T1D pathogenesis.

## Data Availability

The raw data supporting the conclusions of this article will be made available by the authors, without undue reservation.

## Supplementary material

Supplementary Figure 1 Gating strategy used in flow cytometric analysis of the frequency of monocytes. Monocytes were gated based on their morphology (FSC^high^ and SSC^int/high^) and positive expression of CD14 surface marker (double-gating strategy). Next, within that population, we determine the three main subsets of monocytes, including CD14 and CD16 surface expression: CD14++CD16-, classical monocytes; CD14++CD16+, intermediate monocytes; CD14+CD16++, nonclassical monocytes. Sample representative plots for monocytes population, together with necessary unstained and FMO controls are demonstrated.

Supplementary Figure 2 Associations between CD163-expressing monocyte subsets and laboratory parameters in type 1 diabetes, at admission **(A)** and after 2 years of therapy **(B)**. Presentation of exact r values on heat maps with color-indicated strength. Correlation strength was grouped based oncoefficient values: weak (r = 0.20-0.39), moderate (0.40-0.59), and strong (0.60-0.80).

Supplementary Figure 3 Differences in non-classical monocyte frequency in T1D patients, including stratification by diabetic-related autoantibody levels (GADA, IA2A, ICA) into lower/higher **(A)**, and including analysis of activated CD163+ non-classical monocytes **(B)**. The significance levels indicated with asterisks or exact p values: * p<0.05; ** p<0.01; *** p<0.001; **** p<0.0001.

Supplementary Figure 4 Effect of CD163+ monocyte subset levels on the changes within selected parameters in the course of the T1D therapy. Changes in time reported in the context of HbA1c **(A)**, insulin requirement **(B)** and c-peptide **(C)**, within T1D low/high CD163+ monocyte groups. Significant differences were distinguished with asterisks or exact p values (color for time-based changes; black brackets for differences between groups): * - p < 0.05; ** - p < 0.01; *** - p < 0.001; **** p < 0.0001.

Supplementary Figure 5 Effect of lower/higher frequency of CD163-expressing monocytes before therapy on the achievement of normal level of selected parameters **(A)** or remission **(B)**. Differences in selected cytokines plasma levels in type 1 diabetes patients (T1D) and control group (HC) **(C)**. Correlation of monocyte subsets with plasma cytokines (B). Monocyte subsets association with Th17 and Treg cells **(C)**. Significant results indicated with asterisks or exact p values: * p<0.05; ** p<0.01; *** p<0.001; **** p<0.0001. Correlations strength was grouped on the basis of the coefficient values: weak (r = 0.20-0.39), moderate (0.40-0.59) and strong (0.60-0.80).

Supplementary Table 1 Descriptive characteristics of the studied groups - type 1 diabetes pediatric patients (T1D) and healthy controls (HC). Data were presented as a median value with 25^th^ and 75^th^ interquartile ranges in the brackets. **(A)** Median value of diabetes-related clinical parameters within type 1 diabetes pediatric patients (T1D) group together with its reference range in clinical laboratory. Data were presented as a median value with 25^th^ and 75^th^ interquartile ranges in brackets. **(B)**
