# Supplementary material for: Contribution of monocyte subpopulations to the pathogenesis and therapy outcome of type 1 diabetes management in pediatric patients
Source: Front Immunol. 2026 Aug 5;17:1752682. doi: 10.3389/fimmu.2026.1752682 (PMC13485582; doi:10.3389/fimmu.2026.1752682)
Supplement: Supplementary file 1 [file Table1.docx]

**Supplementary Table 1.**

**A.**

|  | **Type 1 diabetes**  **(T1D)** | **Healthy control**  **(HC)** | **p-value** |
| --- | --- | --- | --- |
|  | Median [25^th^ /75^th^ quartile] | | |
| **Patients’ numbers** | 59 | 24 |  |
| **Sex distribution**  **[boys/girls]** | 25/34  [42%/58%] | 11/13  [46%/54%] | p = 0.8106 |
| **Age**  **[years]** | 11.00  [9.0; 14.0] | 14.5  [12.0; 16.0] | p = 0.8973 |
| **Glycemia**  **[mg/dL]** | **430.0**  **[306.50; 519.0]** | **87.0**  **[83.0; 91.0]** | **p < 0.0001** |
| **BMI**  **[kg/m2]** | 16.74  [14.78; 18.60] | 21.40  [18.80; 23.70] | p = 0.8209 |

**B.**

|  | **Type 1 diabetes**  **(T1D)** | **Normal range** |
| --- | --- | --- |
|  | Median [25^th^ /75^th^ quartile] |  |
| **Glycemia**  **[mg/dL]** | 430.0  [306.50; 519.0] | 70-105 |
| **HbA1c**  **[%]** | 11.46  [10.52; 14.0] | < 6.5 |
| **C-peptide**  **[μg/mL]** | 0.52  [0.32; 0.77] | > 1.1 |
| **DIR**  **[insulin/kg/day]** | 0.63  [0.49; 0.71] | < 0.5 |
| **GADA**  **[IU/mL]** | 83.10  [6.20; 289.20] | < 5.0 |
| **IA2A**  **[IU/mL]** | 488.6  [5.0; 1184.20] | < 0.9 |
| **ICA**  **[IU/mL]** | 20.0  [0.0; 40.0] | < 10.0 |
